# Dare to jump: The effect of the new high impact activity SuperJump on bone remodeling. A new tool to maintain fitness during COVID-19 home confinement

**DOI:** 10.5114/biolsport.2022.108993

**Published:** 2021-12-30

**Authors:** Sonya Vasto, Alessandra Amato, Patrizia Proia, Rosalia Caldarella, Cristina Cortis, Sara Baldassano

**Affiliations:** 1 Department of Biological, Chemical and Pharmaceutical Sciences and Technologies; 2 IEMEST, Euro-Mediterranean Institute of Science and Technology, Via Michele Miraglia, 20-90139 Palermo University of Palermo, Italy; 3 Department of Psychological, Pedagogical and Educational Sciences, Sport and Exercise Sciences Research Unit, University of Palermo; 4 Department of Laboratory Medicine, “P. Giaccone” University Hospital, Palermo, Italy; 5 Department of Human Sciences, Society and Health, University of Cassino and Lazio Meridionale

**Keywords:** Bone remodeling, Physical activity, Women, Osteoporosis prevention, Bone homeostasis

## Abstract

SuperJump is a high impact activity performed on an elastic trampoline that mixes aerobic and anaerobic exercises already proposed as home-based activity for preventing a sedentary lifestyle. We determined in a randomized controlled trial whether 20 weeks of SuperJump activity would promote bone formation and reduce resorption in eumenorrheic women. Twenty-four women were randomized to a non-exercise group (control group) or an exercise group that performed SuperJump activity three times a week for 20 weeks. Blood samples were collected in both groups at baseline and at the end of the 20 weeks and compared within and between the groups for C-terminal telopeptide (CTX), a marker of bone resorption, osteocalcin, a marker of bone formation, and the markers of bone metabolism parathyroid hormone (PTH), calcitonin, albumin-adjusted calcium (Aa calcium), vitamin D, phosphate and potassium. After 20 weeks of SuperJump activity, levels of CTX were significantly reduced while levels of osteocalcin were increased. PTH, calcium and potassium were involved in the mechanism of action because PTH was reduced while calcium and potassium were increased. Calcitonin, vitamin D and phosphate levels did not change. These data suggest that SuperJump activity is able to reduce bone resorption and improve bone formation by acting on essential regulators of bone metabolism. They also suggest that SuperJump training may be used as a valuable intervention to prevent the occurrence of osteoporosis in aging because it improves bone homeostasis in favor of bone formation and could counteract a sedentary lifestyle, such as during COVID-19 home confinement, which could itself contribute to the variation of bone metabolism. Trial registration: Clinicaltrials.gov NCT04942691 –retrospectively registered.

## BACKGROUND

Bone mineral density (BMD) reduces across the lifespan [1] and women tend to be more susceptible to bone loss and to the development of osteoporosis than men. Biochemical markers of bone remodeling reflect the cellular activities of bone formation and resorption and can be used to monitor the acute changes in bone remodeling [2]. In fact, they are a useful tool to verify the effects of antiresorptive treatment in osteoporotic patients. They are also used to analyze the influence of external factors, such as exercise and nutrition, on bone remodeling [3, 4].

The bone remodeling is an active and dynamic process and it relies on the correct function of two different cell types responsible for bone metabolism named osteoclasts and osteoblasts. The former are multinucleated cells that destroy the bone matrix, and the latter have osteogenic functions. The interactions between osteoclasts and osteoblasts allow the maintenance of bone integrity. The imbalance between bone resorption and bone formation, in favor of resorption, results in bone loss and deterioration of bone architecture [1]. Exercise has a significant influence on bone health across the lifespan and it seems to be a good candidate for preventive intervention. Several studies have shown that weight-loading exercise increases BMD, positively influences bone development, maintaining strength and reduces falls and risk of fractures.

Jumping, similar to other high-impact loading exercises, has proven to be able to generate a substantial osteogenic response and to increase balance and bone material strength in postmenopausal women [5–7]. Moreover, competitive female trampolinists had greater bone density, area, microarchitecture, and bone strength [8] with respect to other disciplines. The benefits of exercise intervention on bone health can be indicated by the markers of bone turnover other than densitometry [9]. In many studies the levels of bone turnover markers can be indirectly used to detect or monitor the early response of the skeleton to exercise [10, 11].

Finding a new workout program able to improve bone health that positively influences well-being, and with exercise easy to standardize for participants, would be of great interest to prevent osteoporosis and lower the risk for fracture in aging women since adherence and compliance to the treatment of osteoporosis have decreased in recent years [12]. This along with the importance of aging in good health highlights the need of identifying new tools for osteoporosis prevention. Therefore, in recent years, the effects of physical activity have received widespread attention due to potential for prevention of fractures and osteoporosis [13].

It is clear that bone turnover may be affected by the type of training. Thus, the mechanical effects of the exercise, the components such as intensity, [14] and the volume modulates bone response [3]. The home confinement carried out during the COVID19 pandemic was found to be one of the most effective means to combat the pandemic but this has invariably altered people’s lifestyle [15]. SuperJump, is a high impact physical activity performed on trampolining that mixes aerobic and anaerobic exercises. SuperJump training session are based on total body exercises performed by jumping to the rhythm of music on trampolining it was also proposed as an effective and fun home-based activity able to counteract the effects of home confinement induced by the situation of the COVID-19 pandemic [16] in order to maintain a healthy status and fit [17] and be satisfied not only at physical level but also at the psychosocial and behavioral level during the period of confinement [18]. The effects of this workout activity on bone turnover and metabolism has not been investigated so far. We hypothesized that a controlled exercise program such as SuperJump would promote bone formation and reduce resorption in eumenorrheic women. We imagined that markers of bone turnover would be influenced by trampolining exercise and would demonstrate changes indicative of bone remodeling in our training groups and that this would impact on the essential regulators of bone metabolism like calciotropic hormones and markers of calcium metabolism. Therefore, the aim of the study was to investigate the effects of SuperJump training for 20 weeks on bone turnover markers and to investigate the mechanism of action in eumenorrheic women.

## MATERIALS AND METHODS

### Study population

**FIG. 1:**
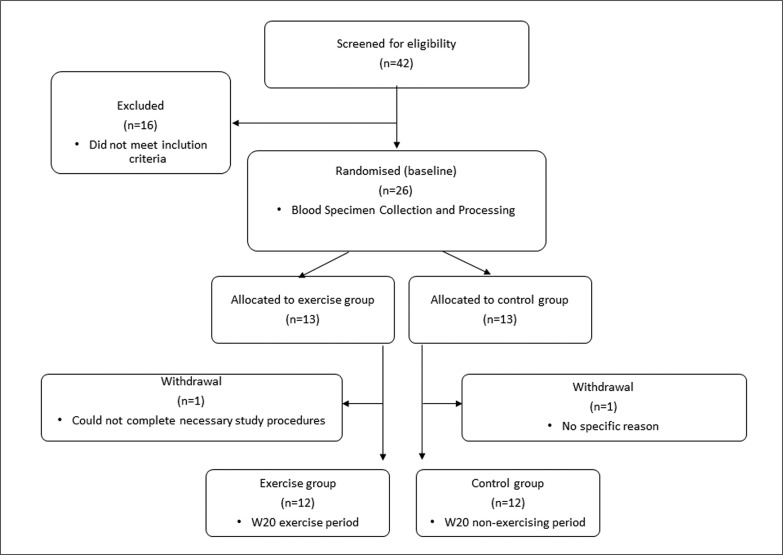
Flow chart of the recruitment and selection process of subjects.

A total of forty-two women volunteered in this randomized-controlled study. The study focused on women because they tend to be more susceptible to bone loss and to the development of osteoporosis compared with men. Participants were asked not to change their daily and diet habits during the study period. They were contacted during the study to remind them not to modify their habits until they had completed the study. Inclusion criteria were 1) women living in Italy 2) age: 18–40 years 3) currently injury free 4) Body mass index between 18.5 and 28 kg/m^2^ 5) self-reported regular and frequent menstrual cycles (menstrual cycle interval between 24 and 35 days). Self-reported menstrual cycle details were assessed using a questionnaire, which included questions about menstrual frequency and length, bleeding length and previous oral contraceptive pill use. Menstrual cycle length was defined as the number of days from the first day of menstruation to the day before the next onset of menstruation. Eumenorrhea was established if menstruation occurred at regular intervals of 24–35 days. Amenorrhoeic women (absence of menstruation for a minimum of 3 repeated months), oligomenorrhea women (menstrual cycles of 36–90 days) and women with short menstrual cycles (menstrual cycles < 24 days) were excluded from participation to ensure that existing reproductive disturbances did not affect the results. Exclusion criteria were 1) bone fracture within the previous year, 2) self-reported long (> 35 days) or short (< 24 days) or irregular menstrual cycles, 3) use of medication or suffering from any condition known to affect bone metabolism, 4) pregnancy, 5) breastfeeding 6) current smokers 7) use of any type of hormonal contraception within the past six months 8) calcium or vitamin D supplementation in the preceding six months 9) participation in moderate and high impact-activity for ≥ 3 h·week before enrolling the study. From a total of forty-two women, sixteen were excluded because of insufficient time to participate or did not meet the inclusion criteria. Twenty-six women were randomized to a non-exercise group (control group) and or an exercise group and performed all the assessments at baseline. One subject in the exercise group and one in the control group discontinued the study without any specific reason. Therefore, twelve women entered in the exercise program and twelve in the control group (Figure 1).

### Experimental design

**FIG. 2:**
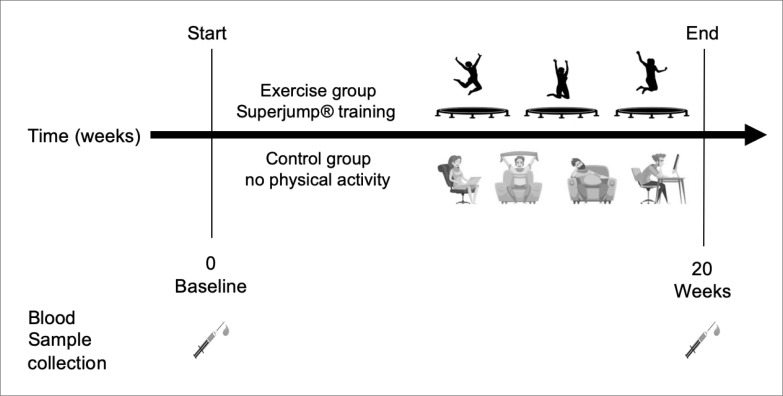
Schematic overview of the study design. Blood samples were collected at time 0 (baseline) and after 20 weeks in the control group and exercise group. In the exercise group, SuperJump training was performed three times a week, each session lasting 60 minutes for a total of 20-weeks. The control group not performed physical activity.

Participants attended the first visit at the onset of menstruation, which indicated the first day of the experimental study. They completed a habitual dietary intake assessment and underwent anthropometric measurement. The blood sample (baseline sample-BASE) was collected prior to the experimental condition start. The control group not performed physical activity. In the exercise group SuperJump training was performed three times a week, each session lasting 60 minutes for a total of 20-weeks. The second sample of blood was collected, at the onset of menstruation, at the end of the 20-weeks (W20). The experimental design is described in figure 2.

### Anthropometric measurement

Body weight was measured after overnight fast on an electronic scale (Gima 27088; Gima, Italy) calibrated to the nearest 0.1 kg. Barefoot standing height was measured to the nearest 0.1 cm by using a wall-mounted stadiometer (Gima 27335; Gima Italy). The coefficients of variation for serial measurements of weight and height were 0.95 and 0.98 respectively. Body mass index (BMI) was calculated as weight, in kilograms, per standing height, meters, squared. Body composition (Lean mass, Fat mass) was measured by electrical bioimpedance measurement (InBody320 Body Composition Analyzer).

### Blood sample and biochemical analysis

To minimize circadian variation, blood samples were obtained at the same time of day (between 07.30 and 08.15 h) for each woman, after an overnight fast (from 20:00 h the previous evening). Blood samples were collected in specific tubes containing EDTA for plasma and were centrifuged immediately at 1509 × g for 10 min at 4°C. Venous blood was dispensed into serum tubes and allowed to clot at room temperature for 30 min before being centrifuged under the same conditions. Serum samples were analyzed for Osteocalcin (Cat. Number 12149133122), PTH (Cat. Number 11972103122), CTX (Cat. Number 11972308122), calcitonin (Cat. Number 06445853190), Vitamin D (Cat. Number 07464215190) by using electro-chemiluminescence immunoassay (ECLIA) (Roche Diagnostics, Burgess Hill, UK) on a Cobas e601 analyzer. The method is FDA-cleared and CE marked. Calcium (Cat. Number 106443), albumin (Cat. Number 05166861), Phosphate (Cat. Number 05171377) and Potassium (Cat. Number 11208764202) were measured in plasma using standard commercial assays supplied by Roche Diagnostics performed on the Roche COBAS c501. Fluctuations in protein concentrations, especially albumin, may cause total calcium concentrations to change independently of the ionized calcium concentration, as such calcium concentrations were ‘corrected’ to give an albumin-adjusted calcium (Aa calcium) value using the following equation: (−0.8 *([Albumin] – 4)) + [Total Ca].

### Exercise program

SuperJump training is an innovative training performed on the elastic minitrampoline (CoalSport, Rome, Italy) [16]. The subject performed 20 weeks of SuperJump training, 3 times per week. All training sessions were led by experienced instructors. Each session lasted sixty minutes and included the following organization: a 5 minutes warm-up with breathing and mobility exercises from upper to lower body, a central phase with jumping exercises involving the total body and 5 minutes of cool-down phase with total body active stretching exercise. The whole training session was carried out on the mini trampoline. The central phase corresponded to a protocol performed in a circuit that is doing multiple sets of exercises, with very short recovery between exercises. Specifically, the circuit consisted of 10 exercises, lasting 50 seconds each, and 10 seconds of active recovery between the exercises. The recovery is also performed on the trampoline. Each circuit is repeated 5 times each training session. The exercises program is shown in table 1.

**TABLE 1:** Exercise training program.

SuperJump workout characteristics	
**Frequency**	3 times/week for 20 weeks
**Intensity**	65–75% of HR_max_
**Session Time**	60 minutes
**Exercise Time**	50 seconds or 25 each side
**Circuit repetitions**	5 times
**Recovery**	10 seconds (jumping on trampoline)
**Exercise type and execution sequence of the exercises**	Isometric lateral raises Biceps Oblique Adductors abductor Triceps Front lifts Split jump alternating drill Pull to the chin Jumping jack single arm Standing russian twist

The exercise sequences, in the central phase, stimulate alternately upper and lower muscle groups to facilitate the redistribution of blood flow in muscle groups far from each other [19].

### Ethics

The study was approved by the University of Palermo ethics committee Palermo 1, Policlinico Giaccone Hospital (2-2020-27). The study was performed in concordance with the Helsinki Declaration. All participants gave oral and written consent to participate. The trial is registered at Clinicaltrials.gov NCT04942691-retrospectively registered.

### Statistics

Based on results of previous studies on exercise and bone remodeling and metabolism [20, 21] the study was powered to detect a change in CTX of 23% (SD 13%) considering a Type I error (α) = 0.05 (two-sided), and Type II error (β) = 0.20 (power is 80%). An *a priori* power calculation determined that eight women were required to achieve 80% power at P < 0.05. Student t tests were used to compare the baseline characteristics of the two groups. Changes between baseline and follow-up within the groups were analyzed by paired t tests; unpaired t tests were used to compare these differences in the two groups.

## RESULTS

### Participant characteristics

Table 2 shows anthropometric measurements of the subjects at the baseline and after 20 weeks.

**TABLE 2:** Characteristics of the subjects measured baseline and after 20 weeks (20 W) in the control group and exercise group.

	Control group		Exercise group	

	BASE	20 W	BASE	20 W

	Mean ± SD	Mean ± SD	Mean ± SD	Mean ± SD
**Weight (Kg)**	58.5 ± 9	61.7 ± 10.5	60.3 ± 8.3	59.9 ± 8.5
**Hight (cm)**	161 ± 0.1	160 ± 0.1	162.3 ± 4.7	162.1 ± 4.8
**Arm (cm)**	28.3 ± 2.8	28.4 ± 2.9	26.5 ± 3.1	24.9 ± 6.7
**Waist (cm)**	73.2 ± 6.1	72.9 ± 5.7	71.8 ± 6.3	68.8 ± 3.7
**Hips (cm)**	100.3 ± 6.1	100.1 ± 6.6	99.1 ± 7	95.6 ± 5.3
**LEAN MASS%**	74.4 ± 5.8	76.6 ± 5.2	73.2 ± 5.9	73.7 ± 7.2
**FATTY MASS %**	25.6 ± 5.8	23.9 ± 6.2	26.8 ± 6	26.3 ± 7.2
**BMI (kg/m^2^)**	22.5 ± 2.7	23.7 ± 2.9	22.8 ± 2.4	22.8 ± 2.8

There were no significant changes in body weight, BMI, and lean or fat body mass in the control group compared to the exercise group or within the exercise or the control group neither at the beginning nor at the end of the intervention period.

### Bone turnover markers

**FIG. 3:**
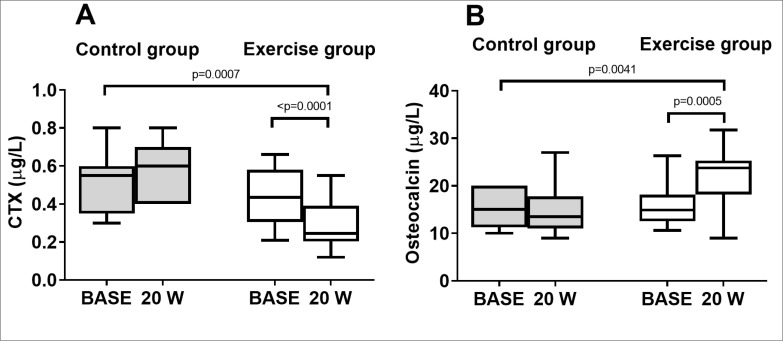
Markers of bone remodeling measured baseline and after 20 weeks in the control group and exercise group. (A) Box and whisker plot of CTX, marker of bone resorption (B) Box and whisker plot of Osteocalcin, marker of bone formation.

In the control group there were no significant changes in the markers of bone remodeling at W20 compared to BASE within the groups (Figure 3). In the exercise group plasma concentrations of the bone resorption marker CTX were significantly reduced at 20 weeks compared to BASE within the groups (figure 3 A). CTX concentrations at W20 decreased by 34% from BASE (0.44 ± 0.1 *vs* 0.29 ± 0.1 μg/L). In addition, the bone formation marker Osteocalcin was significantly increased at W20 compared to BASE (16.2 ± 5 vs 22.2 ± 6 μg/L). Specifically, in the exercise group Osteocalcin increased by 37% at W20 compared to BASE (figure 3 B). The levels detected were within the normal range. The comparison between the groups (Control vs Exercise) showed that there were significant changes in the markers of bone remodeling after 20 weeks of training in the exercise group compared to the control group (Figure 3).

### Markers of bone metabolism

**FIG. 4:**
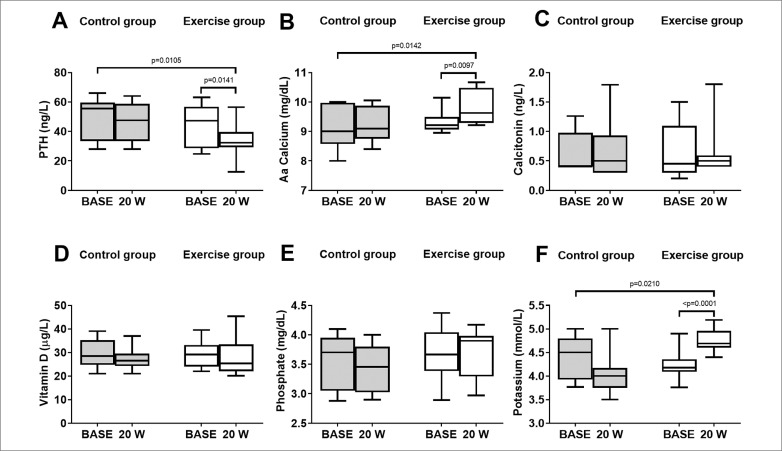
Markers of bone metabolism measured baseline and 20 weeks in the control group and exercise group. (A) Box and whisker plot of PTH (B) Box and whisker plot of Aa Calcium (C) Box and whisker plot of Calcitonin (D) Box and whisker plot of Vitamin D (E) Box and whisker plot of Phosphate (F) Box and whisker plot of Potassium.

In the control group there were no significant changes in the markers of bone metabolism at 20 weeks compared to baseline within the groups (Figure 4). In the exercise group PTH concentrations decreased with time. PTH from BASE was significantly different to W20 within the groups (44 ± 15 vs 34 ± 11 ng/L) (figure 4 A). Specifically, PTH concentrations at W20 decreased by 23% from BASE. Moreover, Aa calcium significantly increased at W20 compared to BASE (Figure 4B). Calcitonin, vitamin D, and phosphate concentrations did not differ between baseline and W20 (Figure 4 C-E). The plasma concentrations of potassium were significantly increased at 20 weeks compared to BASE (figure 4 F). The comparison between the two groups (Control vs Exercise) showed that there were significant changes in the PTH, Aa calcium and potassium after W20 in the exercise group compared to the control group (Figure 4 A, B, F). The levels detected were within the normal range. Calcitonin, vitamin D, and phosphorus were not affected by the 20 weeks of training in the exercise group compared to the control group (Figure 4 C-E).

## DISCUSSION

We investigated whether a new workout activity with high impact, performed on a mini elastic trampoline, called SuperJump, could influence bone metabolism in a short-term exercise intervention in eumenorrheic women. The study suggests that this training program exerts osteogenic action and highlights the importance of high impact physical activity in eumenorrheic women as an essential tool for osteoporosis prevention. In fact, in the study there were no changes in the markers of bone remodeling CTX and Osteocalcin as well as bone metabolism (PTH, ACa, Potassium, Calcitonin, Vitamin D, Phosphate) after 20 weeks in the control group. Thus, the main findings were 1) significant increase in bone formation and decrease in bone resorption following 20 weeks of SuperJump workout class 2) involvement of PTH, calcium and potassium in the mechanism of action.

The study showed that 20 weeks of SuperJump exercise program, three times per week for 60 minutes lasting, induced a significant reduction in the marker of bone resorption CTX and an increase in the marker of bone formation osteocalcin in eumenorrheic women. We can suppose that the decrease in CTX and increase in Osteocalcin we observed might be beneficial for the bone turnover if the levels are maintained within the normal physiological range otherwise it could compromise bone health. In fact, bone formation and resorption are coupled processes. However, a previous study showed that one month of moderate physical activity increases bone formation but does not affect bone resorption in healthy premenopausal women [22]. Thus, further studies are necessary to better understand the influence and the potential beneficial effect of physical activity on bone remodeling in healthy premenopausal women.

Previous studies conducted in competitive female trampolinists have reported health benefits on bone, in particular, competitive female trampolinists have greater bone density, area and microarchitecture [8]. Moreover, the high-impact mechanical loading of jumping exercise for 3 months increased bone material strength index (BMSi) in postmenopausal woman, suggesting that exercise loading impact on bone by regulating bone material proprieties [7]. However, it is important to highlight that the response of the bone appears to be exercise modality, intensity and age specific [23]. In fact, no effects on BMD were reported after 12-week exercise intervention on a mini-trampoline but the markers of bone turnover were not measured. However, the intervention was highly effective in improving balance and functional mobility, strength, gait performance and fear of falling in patients with osteopenia [6]. The exercise program was different to our training program as well as the time of the intervention that was shorter (12 *vs* 20 weeks). Also, the target of the participants was different. In fact, the intervention was conducted in older women with osteopenia while in our study the target population were eumenorrheic women. We don’t know if the SuperJump exercise program, with small modifications to adapt to an older group, could have an impact on bone metabolism or BMD in aging subjects and future studies to investigate it would be of interest. In fact, previous studies have shown that already 6 to 10 weeks of aerobic group-based exercises were able to reduce bone resorption and increase formation in post-menopausal women [9, 10]. However, we focused on eumenorrheic women because although an important goal is to assess efficacy in population most at risk of fracture, it is also of interest to verify the potential preventive effect of a specific physical activity. Quantifying the osteogenic potential of an exercise regimen would be beneficial to assist in developing programs that could serve to promote/maintain/reduce the rate of bone loss during the whole life to prevent injuries and delay sarcopenia that, especially in aging, are a significant burden in terms of cost and personnel.

We investigated the effects on bone remodeling because it is a useful tool not only to monitor the acute effects of exercise on bone health but also to investigate the action mechanism of exercise-induced changes in bone mass [3]. Thereafter, we attempted to provide information about action mechanism of the osteogenic response that accompanies the exercise intervention. In our study, it was observed a significant decrease in PTH level after 20 weeks of SuperJump training whereas elevations in PTH levels are normally associated with increased bone resorption and reductions are associated with bone formation [24]. Our study suggests that this type of high impact activity, by reducing basal PTH level, might have a positive influence on mineral metabolism, particularly by increasing calcium and/or on bone turnover in eumenorrheic women by increasing bone formation and by reducing bone resorption. This is, also, in accordance with previous research, in which pre-menopausal women showed decrease in basal PTH concentrations after high impact exercise training performed for 6 to 12 months [25]. However, the underlying mechanisms influencing PTH release in response to exercise are not well understood. In fact, differently to our study, PTH was increased after 6 weeks of endurance training in elderly men [26]. However, in this study PTH level was measured immediately (< 1 min) after the end of the exercise and the participants were elderly men. Thus, the differences with our study are probably due to the analysis of PTH levels on the acute effects of exercise rather than the gender. In fact, several studies have documented PTH levels decrease below baseline hours after exercise [27, 28]. PTH concentration increases transiently after jumping exercises and aerobic but not after acute resistance exercise [27–30] although studies found that the concentration decreased 2 h post exercise [31, 32]. However, our data support the idea that this type of regular impact training by reducing basal PTH level acts on bone leading to osteogenic effects.

The main action of PTH is to ensure optimal plasma levels of calcium, and it is under strict feedback regulation [25]. Therefore, PTH response would be expected to mediate variation in systemic calcium concentration [29–31]. In agreement with this regulatory mechanism the reduction in PTH secretion was accompanied by a significant increase in Aa calcium level after 20 weeks of SuperJump training. Moreover, it was observed a significant increase in potassium concentration, supporting the positive influence of SuperJump exercise on bone homeostasis. In vitro studies showed that low potassium concentration is able to stimulate bone resorption and interventional studies in humans showed that low potassium concentration induces calcium excretion that in turn impacts on bone remodeling [33, 34]. Thus, both calcium and potassium seem to be involved in the action mechanism that improves bone turnover following 20 weeks of SuperJump training. Calcitonin also impacts on calcium homeostasis since its production is regulated by increased calcium level. The pharmacological function of calcitonin is to inhibit bone resorption through lowered levels of circulating calcium while the physiological role of calcitonin remains unclear. It is known that calcitonin plays a significant role in protecting the skeleton under circumstances of calcium stress [35, 36]. In our study calcitonin was not affected by SuperJump training ruling out its involvement in the action mechanism.

Recent studies suggest an inverse correlation between Vitamin D and PTH in exercising subjects. In fact, during a 32-week training program involving military recruits, there was a high frequency of stress fractures that was associated with poor vitamin D and increased circulating concentrations of PTH [37]. In our case, this study did not observe changes in the vitamin D concentration after 20 weeks of exercise. Thus, vitamin D seems not involved in the observed effects. Phosphate is one of the most abundant minerals in the body, and its blood levels are regulated by a complex set of processes involving not only the skeleton but also the intestine and kidneys. PTH directly causes phosphate resorption from bone and decreases its reabsorption in the proximal tubule, and indirectly by stimulating the production of calcitriol [38]. However, similar to Vitamin D, no changes in phosphate levels were observed after 20 weeks of SuperJump training in the woman.

The presence of a control group allows to exclude changes in marker levels due only to seasonal variation [39] and confirms that the positive effects on bone turnover in the exercise group were a result of exercise. The presence of a class activity group creates social interaction and makes learning exercise easy by gaining support from the peers [40, 41]. The presence of the instructor minimizes the problems with recollection of the exercise program, loss of motivation and skewed image reported for how well participants have performed the exercise because the instructor controlled the adherence to the exercise and stimulated the compliance.

## CONCLUSIONS

In conclusion, 20 weeks of Superjump exercise in eumenorrheic women reduced bone resorption and increased formation. PTH, calcium and potassium seem to play a role in the osteogenic effects. Thus, Superjump exercise might be used as a tool for prevention of osteoporosis in middle age women proposing itself as a valid training alternative to be carried out even at home as during the restrictions induced by the COVID-19 pandemic.

## Data Availability

The datasets during and/or analyzed during the current study are available from the corresponding author on reasonable request.
